# Factors Associated with COVID-19 Vaccine Hesitancy

**DOI:** 10.3390/vaccines9030300

**Published:** 2021-03-22

**Authors:** Patricia Soares, João Victor Rocha, Marta Moniz, Ana Gama, Pedro Almeida Laires, Ana Rita Pedro, Sónia Dias, Andreia Leite, Carla Nunes

**Affiliations:** 1 Comprehensive Health Research Center, Universidade NOVA de Lisboa, Campo Mártires da Pátria 130, 1169-056 Lisboa, Portugal; jv.rocha@ensp.unl.pt (J.V.R.); am.moniz@ensp.unl.pt (M.M.); ana.gama@ensp.unl.pt (A.G.); laires.pedro@gmail.com (P.A.L.); rita.pedro@ensp.unl.pt (A.R.P.); sonia.dias@ensp.unl.pt (S.D.); andreia.leite@ensp.unl.pt (A.L.); CNunes@ensp.unl.pt (C.N.); 2 NOVA National School of Public Health, Public Health Research Center, Universidade NOVA de Lisboa, Av. Padre Cruz, 1600-560 Lisboa, Portugal

**Keywords:** COVID-19, vaccination, vaccine hesitancy

## Abstract

It is critical to develop tailored strategies to increase acceptability of the COVID-19 vaccine and decrease hesitancy. Hence, this study aims to assess and identify factors associated with COVID-19 vaccine hesitancy in Portugal. We used data from a community-based survey, “COVID-19 Barometer: Social Opinion”, which includes data regarding intention to take COVID-19 vaccines, health status, and risk perception in Portugal from September 2020 to January 2021. We used multinomial regression to identify factors associated with intention to delay or refuse to take COVID-19 vaccines. COVID-19 vaccine hesitancy in Portugal was high: 56% would wait and 9% refuse. Several factors were associated with both refusal and delay: being younger, loss of income during the pandemic, no intention of taking the flu vaccine, low confidence in the COVID-19 vaccine and the health service response during the pandemic, worse perception of government measures, perception of the information provided as inconsistent and contradictory, and answering the questionnaire before the release of information regarding the safety and efficacy of COVID-19 vaccines. It is crucial to build confidence in the COVID-19 vaccine as its perceived safety and efficacy were strongly associated with intention to take the vaccine. Governments and health authorities should improve communication and increase trust.

## 1. Introduction

By the first week of January 2021, with more than 89 million COVID-19 cases confirmed and almost 2 million COVID-19 confirmed deaths worldwide [1], the start of the COVID-19 vaccination was a beacon of hope for normal life to return. However, they prompted discussions on vaccination hesitancy as a vaccination program’s success will depend on uptake among the population [2].

Vaccine hesitancy is defined as the delay in acceptance, reluctance, or refusal of vaccination despite the availability of vaccination services [2,3]. It has been identified by the World Health Organization (WHO) as one of the top 10 threats to global health in 2019 [3]. Vaccine hesitancy results from a complex decision-making process, influenced by a wide range of contextual, individual and group, and vaccine-specific factors, including communication and media, historical influences, religion/culture/gender/socioeconomic, politics, geographic barriers, experience with vaccination, risk perception, and design of the vaccination program [2]. A large-scale retrospective study combined previously published data from 284,381 individual responses to the Vaccine Confidence Index survey conducted in 149 countries between 2015 and 2019 to measure confidence (and, conversely, hesitancy) and identified that confidence in vaccines remained low across Europe compared to other continents [4]. While being male, having fewer years of education, or being part of minority religious groups were associated with decreased chances of vaccine uptake, confidence in the importance of vaccines, information-seeking behaviours, and trust in healthcare workers were associated with increased chances [4].

Some studies explored COVID-19 vaccine acceptance and their determinants using surveys between March and December 2020. These were conducted in China [5], Indonesia [6], Italy [7], Ireland [8], Japan [9], United Kingdom [10], and the United States [11,12,13,14,15,16]. In addition, multicountry surveys have been undertaken in 7 European countries [17], 18 Arab countries [18], and 19 countries worldwide [19]. A rapid systematic review identified 23 articles that used surveys to assess confidence in and receptivity for COVID-19 vaccines [20]. Several of these studies indicated that older individuals and those with higher income and education levels were more likely to accept a vaccine [11,12,14,18,19,20]. Other factors associated with more willingness to be vaccinated included higher trust in government decisions [19], having been vaccinated against the flu in the previous year [5,12,14], perceiving COVID-19 as a threat to self [6,9,12,15] and the community [9,12,13], having health insurance [12], knowing people who were infected by COVID-19 [12,14], and being a health professional [6].

Most of these studies on COVID-19 vaccine acceptance were conducted before October 2020 when the vaccine was still far away. Authors recognised that acceptance was mostly assessed using hypothetical questions as there were still no vaccines developed [6,11,12]. Furthermore, the pandemic situation and interventions in place could have affected participants’ response at the time of the survey and thus the overall findings [7,11,19]. As vaccination of the general population starts, an in-depth understanding of the current situation and determinants of vaccine hesitancy is crucial to ensure proper action to achieve sufficient vaccination coverage, allowing attainment of herd immunity.

Portugal has a higher vaccine coverage than the EU average for several vaccines [21,22]. Vaccination coverage is highly influenced by its acceptability and the overall perception of its benefit/safety profile. Vaccine hesitancy is a barrier to achieve high vaccination coverage against infectious diseases. Therefore, understanding its determinants is necessary to aid acceptability and tackle vaccine hesitancy [23] and consequently achieve high coverage for this new vaccine. Hence, this study aims to assess and identify factors associated with COVID-19 vaccine hesitancy in Portugal.

## 2. Methods

### 2.1. Study Design

Data from the community-based survey “COVID-19 Barometer: Social Opinion” was used [24]. The survey contains data on perception of risk, health status, social experiences, and use of health services during the COVID-19 pandemic. It is an open cohort study, where participants can answer the questionnaire once or more than once. Data collection is still ongoing, with more than 185,000 answers by 8 January 2021. The questionnaire is flexible to adjust rapidly to the dynamic situation we are currently experiencing. Our study started on 29 September 2020, when the questions about vaccination were added, and ended on 8 January 2021, almost two weeks after the beginning of COVID-19 vaccination in Portugal [25]. Only the question about the perception of information provided by health authorities was added during the study period, on 24 October 2020. We excluded participants who were not living in Portugal. We decided to do a cross-sectional study using the last response per participant because the number of individuals who answered almost every fortnight was low (*n* = 48).

### 2.2. Outcome

At the end of September, two questions about vaccines were introduced in the questionnaire. One of the questions related to intention of the participant to take the vaccine (“By the time a vaccine against COVID-19 is available, what is your intention for taking it?”). The other question concerned trust in the vaccine (“What is your level of confidence in the COVID-19 vaccines that are being developed, in terms of safety and efficacy?”). The outcome for this analysis corresponds to the question regarding intention of the participant to take a vaccine. The participant can answer: “Yes, as soon as the vaccine is available”, “Yes, but I will wait some time”, “Yes, but I will wait a long time”, and “No, I will not take the vaccine”.

The outcome was categorized into three categories: “Yes”, corresponding to participants that will take the vaccine as soon as possible; “Wait”, corresponding to participants who will wait for some time or a long time before taking the vaccine, serving as a proxy for delay; and “No”, corresponding to participants who have no intention of taking the COVID-19 vaccine, serving as a proxy for refusal.

### 2.3. Independent Variables

The variables considered as potential determinants of vaccine hesitancy were grouped following the framework developed by the Working Group on Vaccine Hesitancy as contextual, individual and group, and vaccine-specific influences [2]. Due to the current situation, we added a fourth category regarding disease-specific variables. We also created a variable concerning the period of the questionnaire, which was intended to capture possible influences of the information concerning the safety and efficacy of COVID-19 vaccines and was divided into before (September and October) or after (November to January) this release [26,27]. Table 1 presents the variables considered for this analysis.

### 2.4. Statistical Analysis

Although the outcome of interest is an ordinal variable, the assumption of proportional odds was not upheld (data not shown). Hence, a multinomial model was fitted with a nominal dependent variable with three categories (“Yes”, “Wait”, and “No”) representing intention to vaccinate. The independent variables considered were selected a priori and grouped based on the vaccine hesitancy determinant matrix (Table 1). Groups included contextual, individual and group, and vaccine-specific influences [2]. Owing to specificities of the pandemic situation, we added a fourth group of disease-specific determinants. Odds ratio (ORs) and corresponding 95% confidence intervals (CI) were estimated for all variables. ORs were adjusted for age, gender, education, and period of the questionnaire.

Due to the high proportion of participants with a university degree, a sensitivity analysis was performed, stratifying the analysis by education. We aggregated confidence in the COVID-19 vaccine into very confident and confident versus not very confident and not confident due to quasi-complete separation between the outcome and confidence in the COVID-19 vaccine. We did the same for the variable concerning adequacy of the government’s measures (very adequate and adequate versus not very adequate and not adequate) due to the low numbers in the sensitivity analysis.

All statistical analyses were performed using R 4.0.2 [28] using the package MASS and nnet [29] for the regressions and the package ggplot2 [30] for the figures.

## 3. Results

We included a total of 1943 individuals, of which 686 (35.3%) would take the vaccine as soon as possible, 1079 (55.5%) would wait before taking the vaccine (869 (44.7%) would wait some time and 210 (10.8%) would wait a long time), and 178 (9.2%) would not take the vaccine. Table 1 presents the characteristics of the sample. Overall, more women (71.1%) and individuals with a university degree (76.2%) answered the questionnaire. More participants without a university degree answered that they would not take the vaccine than participants with a university degree (13.9% versus 8.2%). The situation was reversed for individuals who would wait before taking the vaccine, with a higher percentage for individuals with a university degree (55.7% versus 50.6%). Individuals who would take the vaccine as soon as possible had a mean ± standard deviation age of 47.7 ± 13.0 years, while individuals who would wait and would not take the vaccine had a younger mean age of 45.4 ± 12.1 and 44.9 ± 10.2, respectively (Table 2).

Crude ORs and adjusted OR (aOR) with their respective 95% CI can be found in the Supplementary Material. The sensitivity analysis can also be found in the Supplementary Material.

### 3.1. Determinants of Vaccine Hesitancy: Contextual Influences

**Figure 1:**
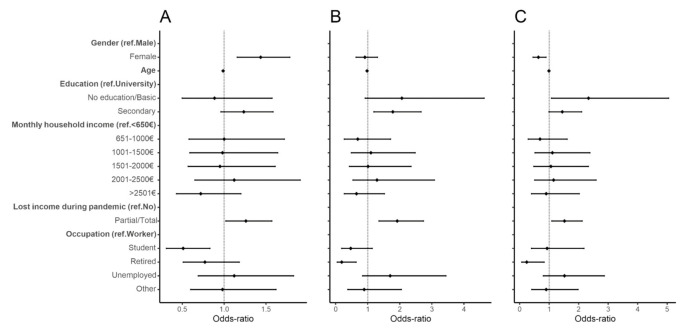
Forest plot of vaccine hesitancy for contextual influences. Adjusted odds-ratio (adjusted for gender, age, education, and period of questionnaire) and the respective 95% confidence intervals are denoted by black dots and black lines, respectively. (**A**) Individuals who would wait to take the vaccine compared to individuals who would take the vaccine; (**B**) individuals who would not take the vaccine compared to individuals who would take the vaccine; (**C**) individuals who would not take the vaccine compared to individuals who would wait to take the vaccine.

Older individuals were more likely to take the vaccine as soon as possible (Wait versus Yes, aOR: 0.99, 95% CI: 0.98, 0.99; No versus Yes, aOR: 0.98, 95% CI: 0.97, 0.99) (Figure 1 and Table S1). Individuals with secondary education or individuals without education or with a basic education were more likely not to take the COVID-19 vaccine than individuals with a university degree (No versus Yes, aOR: 1.78, 95% CI: 1.19, 2.66; No versus Wait, aOR: 2.33, 95% CI: 1.08, 5.05). Females had higher odds of delaying vaccine intake than males (Wait versus Yes, aOR: 1.44, 95% CI: 1.16, 1.78; No versus Wait, aOR: 0.63, 95% CI: 0.45, 0.89). A higher odds of delay and refusal was also found for individuals who lost income during the pandemic compared to those who did not (Wait versus Yes, aOR: 1.26, 95% CI: 1.02, 1.57; No versus Yes, aOR: 1.92, 95% CI: 1.35, 2.74; No versus Wait, aOR: 1.52, 95% CI: 1.09, 2.12). Students had lower odds of delay compared to workers (Wait versus Yes, aOR: 0.51, 95% CI: 0.31, 0.83), while retired individuals had lower odds of refusal compared to workers (No versus Yes, aOR: 0.19, 95% CI: 0.05, 0.64; No versus Wait, aOR: 0.24, 95% CI: 0.07, 0.83). Regarding the monthly household income, no association was found (Figure 1 and Table S1).

Restricting the analysis for individuals with a university degree, age, gender, and retirement were no longer associated with refusal to take the COVID-19 vaccine (Table S3). For individuals without a university degree, the results were no longer significant for gender, age, income loss, and occupation (Table S2). However, although monthly household income was not significantly associated with intention to take the vaccine, different results were found in the sensitivity analysis. Participants without a university degree and higher household income had lower odds of refusal and delay than households with less than 650 € per month income (Table S2). In contrast, participants with a university degree and higher household income had higher odds of delay than participants with household income less than 650 € per month (Table S3). However, these results were not consistent among all income classes.

### 3.2. Determinants of Vaccine Hesitancy: Individual and Group Influences

**Figure 2:**
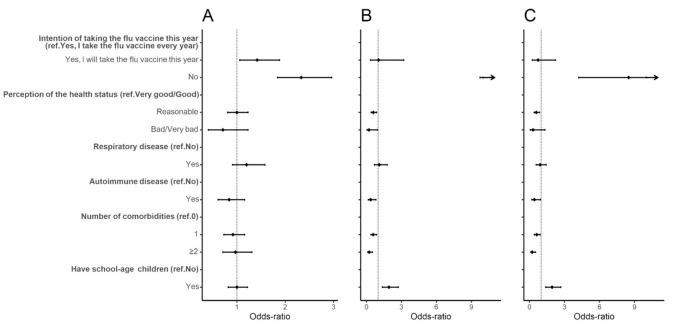
Forest plot of vaccine hesitancy for individual and group influences. Adjusted odds-ratio (adjusted for gender, age, education, and period of questionnaire) and the respective 95% confidence intervals are denoted by black dots and black lines, respectively. Forest plot confidence intervals and estimates were cut off at 10. (**A**) Individuals who would wait to take the vaccine compared to individuals who would take the vaccine; (**B**) individuals who would not take the vaccine compared to individuals who would take the vaccine; (**C**) individuals who would not take the vaccine compared to individuals who would wait to take the vaccine.

An increased odds of delay was found for individuals who would not take the flu vaccine this year compared to those who would take it (Wait versus Yes, aOR: 2.33, 95% CI: 1.84, 2.96). This factor was also strongly associated with higher odds of refusal (No versus Yes, aOR: 19.81, 95% CI: 9.74, 40.30; No versus Wait, aOR: 8.50, 95% CI: 4.22, 17.11) (Figure 2 and Table S1). Individuals who perceived their health status as reasonable compared to good or very good had lower odds of refusal (No versus Yes, aOR: 0.59, 95% CI: 0.40, 0.87; No versus Wait, aOR: 0.59, 95% CI: 0.41, 0.86). Similarly, individuals with comorbidities had lower odds of refusal than individuals without comorbidities (Figure 2 and Table S1). A higher odds of refusal was also found for individuals with school-age children compared to those without (No versus Yes, aOR: 1.93, 95% CI: 1.37, 2.73; No versus Wait, aOR: 1.94, 95% CI: 1.39, 2.69).

Similar results were found in the sensitivity analyses for individuals with a university degree (Table S3). In contrast, for individuals without a university degree, the results lost significance for those with school-age children. Higher odds of delay were found for individuals without a university degree who perceived their health status as bad or very bad compared to good or very good (Wait versus Yes, aOR: 0.42, 95% CI: 0.18, 0.94) (Table S2).

### 3.3. Determinants of Vaccine Hesitancy: COVID-19 Influences

**Figure 3:**
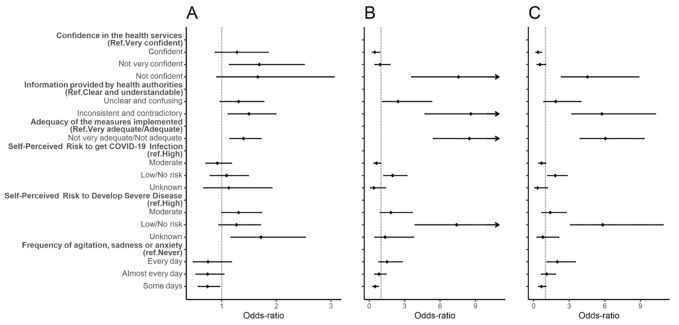
Forest plot of vaccine hesitancy for COVID-19-specific factors. Adjusted odds-ratio (adjusted for gender, age, education, and period of questionnaire) and the respective 95% confidence intervals are denoted by black dots and black lines, respectively. Forest plot confidence intervals and estimates were cut off at 10. (**A**) Individuals who would wait to take the vaccine compared to individuals who would take the vaccine; (**B**) individuals who would not take the vaccine compared to individuals who would take the vaccine; (**C**) individuals who would not take the vaccine compared to individuals who would wait to take the vaccine.

An increased odds of refusal was found for individuals who found the measures implemented by the government to be inadequate (No versus Yes, aOR: 8.49, 95% CI: 5.44, 13.25; No versus Wait, aOR: 6.04, 95% CI: 3.93, 9.30) and for individuals who found the information provided by health authorities to be inconsistent and contradictory as opposed to those who found the information clear and understandable (No versus Yes, aOR: 8.61, 95% CI: 4.73, 15.68; No versus Wait, aOR: 5.75, 95% CI: 3.23, 10.26). These factors were also associated with delay (Figure 3 and Table S1). Lack of confidence in the health service response to the pandemic presented a higher odds of refusal compared to individuals who were very confident with the response (No versus Yes, aOR: 7.56, 95% CI: 3.59, 15.92; No versus Wait, aOR: 4.54, 95% CI: 2.33, 8.87).

Those perceiving a low/nonexisting risk of infection had higher odds of refusal compared to those perceiving their risk as high (No versus Yes, aOR: 1.98, 95% CI: 1.23, 3.20; No versus Wait, aOR: 1.83, 95% CI: 1.18, 2.84), while those who perceived a moderate risk of infection had lower odds of refusal (No versus Yes, aOR: 0.61, 95% CI: 0.39, 0.96). Individuals perceiving their risk of developing complications following COVID-19 infection as low/nonexisting had higher odds of refusal than those perceiving a high risk (No versus Yes, aOR: 7.41, 95% CI: 3.89, 14.11; No versus Wait, aOR: 5.82, 95% CI: 3.10, 10.91) (Figure 3 and Table S1).

Individuals who felt agitated, sad, or anxious due to the physical distancing measures on some days had lower odds of refusal than individuals who never had those feelings (No versus Yes, aOR: 0.49, 95% CI: 0.31, 0.78). However, individuals who felt agitated, sad, or anxious due to the physical distancing measures every day had higher odds of refusal than those who never had those feelings (No versus Wait, aOR: 2.00, 95% CI: 1.13, 3.51) (Figure 3 and Table S1).

No significant association was found for individuals with a university degree between the perceived risk of infection and intention to vaccinate (Table S3). Results were not consistent for individuals without a university degree, as the perception of the government’s measures and the quality of the information provided by health authorities were no longer significantly associated with delay to take the vaccine (Table S2).

### 3.4. Determinants of Vaccine Hesitancy: COVID-19 Vaccine Influences

An increased odds of delay was found for individuals who have little to no trust in the COVID-19 vaccines being developed than those who trust them (Wait versus Yes, aOR: 9.94, 95% CI: 7.48, 13.20). This factor was also strongly associated with higher odds of refusal (No versus Yes, aOR: 109.69, 95% CI: 57.38, 206.69; No versus Wait, aOR: 11.04, 95% CI: 6.01, 20.28) (Table S1). Questionnaires answered before the release of information regarding the safety and efficacy of COVID-19 vaccines were also strongly associated with higher odds of delay and refusal compared to those who answered after the release of this information (Wait versus Yes, aOR: 2.05, 95% CI: 1.68, 2.50; No versus Yes, aOR: 4.69, 95% CI: 3.21, 6.86; No versus Wait, aOR: 2.29, 95% CI: 1.59, 3.30) (Table S1).

These factors remained significant in the sensitivity analyses (Tables S2 and S3).

## 4. Discussion

This study found that 35% of the participants would take the vaccine as soon as possible, 56% would wait before taking the vaccine, and 9% would not take the vaccine.

We found that the following factors were associated with both refusal and delay to take the vaccine: (i) contextual factors: younger age and loss of income during the pandemic; (ii) individual and group factors: no intention of taking the flu vaccine this year; (iii) COVID-19 influences: low confidence in the health service response during the pandemic, worse perception of the adequacy of measures implemented by the government, and perception that the information provided by health authorities during the pandemic was inconsistent and contradictory; and (iv) COVID-19 vaccine-specific factors: low confidence in the COVID-19 vaccines being developed and answering the questionnaire before the release of information regarding the safety and efficacy of COVID-19 vaccines.

Specifically, for intention of delaying intake of the COVID-19 vaccine, we found increased odds for the following factors: (i) contextual factors: being female; (ii) individual and group factors: intention of taking the flu vaccine this year; and (iii) COVID-19 influences: unclear perceived risk of developing severe disease following COVID-19 infection. Decreased odds were found for the following: (i) contextual factors: being a student. For intention of refusing intake of the COVID-19 vaccine, we found increased odds for the following factors: (i) contextual factors: having lower levels of education; (ii) individual and group factors: having school-age children; and (iii) COVID-19 influences: low or nonexistent perceived risk of getting COVID-19 infection or developing severe disease following the infection. Meanwhile, decreased odds were found for the following: (i) contextual factors: being retired and (ii) individual and group factors: perception of worse health status and having comorbidities.

Our findings showed a considerable level of COVID-19 vaccine hesitancy in Portugal, higher than that found in previous studies [5,6,8,9,10,11,12,15,17]. The proportion of individuals who would refuse to take the vaccine was similar to the prevalence reported by other studies, which has mainly ranged between 4% and 14.2% [5,7,11,15,16,17,19], although one study in the USA reported that 31% of the participants were unwilling to take the COVID-19 vaccine [12] while another reported that 20% of the participants were unwilling to take the COVID-19 vaccine due to the collateral effects and 14% because they do not need the vaccine [14]. However, we also found that 56% of the participants would wait some time or a long time to take the COVID-19 vaccine. To the best of our knowledge, only two studies have explored delay to take the vaccine. Wang et al. [5] found that 43.7% of individuals would delay vaccination until vaccine safety and efficacy were proven, and Mercadante et al. [14] found that 42% of individuals would wait a few months before taking the vaccine. Other studies have explored the uncertainty regarding intention to take the COVID-19 vaccine, which ranged between 12% and 42% [5,9,10,11,14,15,16,17]. However, these studies examined uncertainty and not hesitancy. While there is no previously established data on the threat to herd immunity, and it is not entirely clear how long individuals would wait, such high frequency of delay in getting the vaccine is worrisome and requires action.

We found several factors associated with vaccine hesitancy. Refusal and delay of taking the COVID-19 vaccine were higher before information on the safety and efficacy of the COVID-19 vaccine was released, suggesting that individuals may be reacting to information made available and exemplifying how hesitancy is a complex, time-dependent construct that is influenced by several factors. A systematic review and meta-analysis study using large, nationally representative samples found divergent results compared to ours. As the pandemic progressed, the percentage of people intending to refuse vaccination increased [31]. However, the analysis only included studies conducted up to October 2020, before the release of information regarding the safety and efficacy of the first COVID-19 vaccine. Another systematic review, which included studies published up to October 2020, found that concerns over vaccine safety and efficacy were among the main reasons for refusal of the vaccine [20]. This is one of the first studies exploring COVID-19 vaccine hesitancy after the release of information regarding the safety and efficacy of COVID-19 vaccines. Our analysis timeframe allowed us to observe a shift in attitude toward COVID-19 vaccination in Portugal, thus demonstrating the time-sensitivity aspect of this construct and how the perception of efficacy and the government’s communication may influence vaccine hesitancy. High efficacy and safety of COVID-19 vaccines may increase confidence in them, hence reducing vaccine hesitancy. The government should continue to update the public about side effects to increase confidence in vaccines. It would also be important to understand how the implementation of public health measures influences vaccine hesitancy. During our study period, several public health measures, namely curfews, were tiered at the municipality level according to the incidence in each municipality in Portugal. We were unable to account for these measures as we did not collect information regarding the municipality of the participantsAdditionally, many countries (especially in Europe) have been facing third waves in early 2021 [1], and nonpharmaceutical government measures have been constantly implemented and lifted. These new developments in the pandemic have certainly affected the rate of vaccine hesitancy. The progress of COVID-19 vaccination coverage has also most likely influenced time variations in vaccine hesitancy in a complex dynamic. Healthcare workers are one of the first groups to receive COVID-19 vaccination; however, previous studies have found conflicting results concerning vaccine hesitancy among healthcare workers for COVID-19 [6,13] and other respiratory diseases [32,33]. Hence, it will be fundamental to continue monitoring COVID-19 vaccine hesitancy over the following months to adjust measures to tackle vaccine hesitancy, thus ensuring adequate vaccination uptake.

Our findings regarding factors associated with delaying or refusing the COVID-19 vaccine are in agreement with previous studies that examined the population in the first semester of 2020. The factors we found to be associated with higher odds of delay and refusal and in agreement with previous findings were not taking the influenza vaccine in the previous season [5,11,14], low or nonexistent perceived risk of getting COVID-19 [5,6,9,12,15,17], and trust in the government [19]. We also found that younger individuals had higher odds of refusal and females had higher odds of delay. Although some studies found higher odds of refusal for younger individuals [5,9,11,13,14,19] and females [5,11,12,18], these findings are inconsistent across the literature, with some studies finding no association [6,7,20] and one study finding men were less likely to take the vaccine [19]. Our study also found that lower trust in/poorer perception of the government and the measures they have implemented, health service response, and information provided by health authorities were associated with delaying or refusing COVID-19 vaccine. Other studies have also reported lack of trust in government/health authorities as factors associated with COVID-19 vaccine hesitancy [11,19]. We found that confidence in the COVID-19 vaccines being developed was associated with higher chances of being vaccinated. Specific vaccine concerns regarding its safety and potential side effects [5,9,12,17,20] are consistently among the main reasons found in the literature for not being vaccinated when the COVID-19 vaccine is ready. We also found that individuals with poorer perception of their health and with comorbidities had lower odds of refusal.

The Working Group on Vaccine Hesitancy discussed that, unlike social determinants of health, vaccine hesitancy determinants, such as education and socioeconomic status, do not have associations in only one direction [2]. Several studies have reported an association between COVID-19 vaccine acceptability and education [5,11,13,19]. We found that individuals with lower education levels were more likely not to take the vaccine than individuals with a university degree. Income loss was also associated with higher odds of refusal. Although income loss has not been previously studied, some studies found that intention to take the vaccine was different across different ethnicities and economic situations [10,12], suggesting that different socioeconomic backgrounds may have different views regarding COVID-19 vaccination.

When stratifying the analysis by education, we identified varying directions of association regarding income. For participants without a university degree, a higher household income was associated with lower odds of refusal and delay, while for participants with a university degree, it was associated with higher odds of refusal and delay. As these results were not consistent among all income classes, further studies should be conducted to explore the relationship between different factors and their impact on vaccine hesitancy among individuals with different education levels.

Because intention to take the COVID-19 vaccine and its perceived safety and efficacy were strongly associated, it is crucial to build confidence in the vaccine. Interventions should be implemented with different communication techniques to explain the risk of COVID-19 to the population. At the same time, individuals should be educated about herd immunity, vaccine safety, and how vaccines can help people to return to their daily lives. Decreasing vaccine hesitancy will help ensure better vaccine coverage. Opposition to vaccines may influence COVID-19 vaccine hesitancy. Hence, governments and health authorities should enhance efforts to encourage trust in vaccines and reduce misinformation.

Although our results are in agreement with previous studies, our study is exploratory and not representative of the Portuguese population, as indicated by an overrepresentation of women and those with a university degree. We may have overestimated the prevalence of individuals who would delay vaccination as more women would take the vaccine after some time. We conducted a sensitivity analysis to understand how the identified factors differed among different education levels. However, due to the lower number of individuals without a university degree, our analysis may be underpowered, possibly explaining why some factors were not significant in this subgroup. Our study also suffers from sampling bias as responses were limited to internet users and users interested or willing to participate in online studies; thus, vulnerable populations are likely underrepresented. Response and nonresponse bias are also present. Participants may have changed their answers to an answer deemed more socially acceptable, although data collection online should have minimised this effect. In contrast, participants who do not believe in the seriousness of COVID-19 may be less likely to spend time filling a questionnaire regarding the disease, hence underestimating the proportion of individuals who would not take the COVID-19 vaccine and individuals who perceive their risk of getting COVID-19 and complications as low or nonexisting. There is also a possibility of repeated sampling. Although we minimised this issue by creating a unique code combining the birthdates of the participant and their mother, their gender, residence, and education, we were unable to distinguish between people who share a birthday, live in the same region and have the same education, or between individuals that do not provide information about birthdates. Furthermore, individuals may have underreported their diseases as the prevalence of chronic diseases was lower in our database than that reported in the National Health Survey [34].

Nevertheless, to date, this is the most recent study on COVID-19 vaccine hesitancy. Although online questionnaires have limitations, the use of these questionnaires is cost-effective and allows quick monitoring of the behaviours of the population and acceptability of the vaccine through time.

These results have implications for the vaccination plans that started at the end of December 2020. We found subgroups that are more likely to refuse or delay vaccination, thus enhancing the importance of providing information that the public perceives as clear and understandable by different channels. Vaccination intention may also be higher than actual vaccine uptake; thus, vaccine intention and hesitancy should be constantly monitored and evaluated to change strategies as deemed necessary. Targeted information should be released by trusted people, which may vary according to the subgroup.

In conclusion, our data suggest that considerable vaccine hesitancy persists, even with the widespread availability, at least in the developed world, of highly effective and safe immunization strategies. To achieve community-based immunity, over 70% of the population will need to be vaccinated [35,36], more than those who seem to be ready to accept such an intervention at this time according to our data. It will be important to devise strategies to meet this threshold as part of a society-wide response to the pandemic. Questionnaires such as ours could be important tools to measure, in a longitudinal fashion, whether we have been able to do so.

## Figures and Tables

**Table 1:** Vaccine hesitancy determinant matrix recommended by the Strategic Advisory Group of Experts (SAGE) Working Group on Vaccine Hesitancy [2], with the fourth category specific to the COVID-19 disease.

Determinants of Vaccine Hesitancy	Variables
Contextual influences	Gender
	Age
	Education
	Monthly household income
	Partial or total income loss during the pandemic
	Occupation
Individual and group influences	Intention to take the flu vaccine
	Perception of the health status
	Number of comorbidities
	Self-reported diabetes
	Self-reported respiratory disease
	Self-reported autoimmune disease
	Having school-age children
COVID-19 disease-specific	Confidence in the capacity of health services to respond to the pandemic
	View on the information provided by health authorities
	Perception of the adequacy of measures implemented by the government
	Self-perceived risk to get COVID-19 infection
	Self-perceived risk to develop severe disease following COVID-19 infection
	Frequency of agitation, sadness, or anxiety due to the physical distancing measures
COVID-19 vaccine-specific	Confidence in the efficacy and safety of COVID-19 vaccines being developed
	Period of the questionnaire

**Table 2:** Sample characteristics according to contextual, individual and group, and COVID-19 influences.

	Yes (*n* = 686)	Wait (*n* = 1079)	No (*n* = 178)
**Contextual influences**			
**Gender (*n* = 1935)**			
Male	220 (32.3%)	274 (25.5%)	65 (36.5%)
Female	462 (67.7%)	801 (74.5%)	113 (63.5%)
**Age (in years) (*n* = 1943)**			
Mean (SD)	47.7 (13.0)	45.4 (12.1)	44.9 (10.2)
**Education (*n* = 1939)**			
No education/Basic education	24 (3.50%)	28 (2.60%)	10 (5.62%)
Secondary	129 (18.8%)	224 (20.8%)	47 (26.4%)
University	533 (77.7%)	823 (76.6%)	121 (68.0%)
**Monthly household income (*n* = 1766)**			
<650 €	30 (4.73%)	58 (5.88%)	11 (7.59%)
651–1000 €	69 (10.9%)	134 (13.6%)	15 (10.3%)
1001–1500 €	136 (21.5%)	225 (22.8%)	37 (25.5%)
1501–2000 €	107 (16.9%)	175 (17.7%)	27 (18.6%)
2001–2500 €	85 (13.4%)	163 (16.5%)	25 (17.2%)
>2501 €	207 (32.6%)	232 (23.5%)	30 (20.7%)
**Lost of income due to the pandemic (*n* = 1913)**			
No	491 (72.4%)	708 (66.7%)	98 (56.3%)
Partial/Total	187 (27.6%)	353 (33.3%)	76 (43.7%)
**Occupation (*n* = 1943)**			
Worker	519 (75.7%)	865 (80.2%)	145 (81.5%)
Student	40 (5.83%)	51 (4.73%)	8 (4.49%)
Retired	27 (3.94%)	48 (4.45%)	8 (4.49%)
Unemployed	73 (10.6%)	63 (5.84%)	3 (1.69%)
Other	27 (3.94%)	52 (4.82%)	14 (7.87%)
**Individual and group influences**			
**Intention of taking the flu vaccine this year (*n* = 1924)**			
Yes, I take the flu vaccine every year	272 (40.1%)	255 (23.9%)	9 (5.06%)
Yes, I will take the flu vaccine this year	136 (20.0%)	201 (18.8%)	5 (2.81%)
No	271 (39.9%)	611 (57.3%)	164 (92.1%)
**Perception of the health status (*n* = 1941)**			
Very good/Good	393 (57.3%)	642 (59.6%)	127 (71.8%)
Reasonable	263 (38.3%)	408 (37.8%)	48 (27.1%)
Bad/Very bad	30 (4.37%)	28 (2.60%)	2 (1.13%)
**Respiratory disease (*n* = 1893)**			
No	571 (85.1%)	872 (83.0%)	145 (84.8%)
Yes	100 (14.9%)	179 (17.0%)	26 (15.2%)
**Autoimmune disease (*n* = 1893)**			
No	593 (88.4%)	945 (89.9%)	164 (95.9%)
Yes	78 (11.6%)	106 (10.1%)	7 (4.09%)
**Number of comorbidities (*n* = 1893)**			
0	350 (52.2%)	587 (55.9%)	120 (70.2%)
1	218 (32.5%)	317 (30.2%)	43 (25.1%)
≥2	103 (15.4%)	147 (14.0%)	8 (4.68%)
**Have school-age children (*n* = 1937)**			
No	409 (59.7%)	633 (58.8%)	76 (43.2%)
Yes	276 (40.3%)	443 (41.2%)	100 (56.8%)
**COVID-19 influences**			
**Confidence in the capacity of health services to respond to the pandemic (*n* = 1926)**			
Very confident	64 (9.38%)	70 (6.52%)	17 (9.94%)
Confident	420 (61.6%)	609 (56.8%)	53 (31.0%)
Not very confident	174 (25.5%)	347 (32.3%)	46 (26.9%)
Not confident	24 (3.52%)	47 (4.38%)	55 (32.2%)
**View on the information provided by health authorities (*n* = 1401)**			
Clear and understandable	334 (61.9%)	417 (53.4%)	18 (22.5%)
Unclear and confusing	99 (18.3%)	153 (19.6%)	12 (15.0%)
Inconsistent and contradictory	107 (19.8%)	211 (27.0%)	50 (62.5%)
**Perception of the adequacy of measures implemented by the government (*n* = 1907)**			
Very adequate	39 (5.77%)	30 (2.84%)	2 (1.14%)
Adequate	386 (57.1%)	540 (51.2%)	25 (14.2%)
Not very adequate	228 (33.7%)	433 (41.0%)	67 (38.1%)
Not adequate	23 (3.40%)	52 (4.93%)	82 (46.6%)
**Self-perceived risk to get COVID-19 infection (*n* = 1942)**			
High	133 (19.4%)	234 (21.7%)	42 (23.6%)
Moderate	392 (57.2%)	582 (53.9%)	67 (37.6%)
Low/No risk	132 (19.3%)	215 (19.9%)	66 (37.1%)
Not sure	28 (4.09%)	48 (4.45%)	3 (1.69%)
**Self-perceived risk to develop severe disease following COVID-19 infection (*n* = 1940)**			
High	156 (22.8%)	184 (17.1%)	13 (7.30%)
Moderate	242 (35.3%)	382 (35.5%)	33 (18.5%)
Low/No risk	229 (33.4%)	390 (36.2%)	126 (70.8%)
Not sure	58 (8.47%)	121 (11.2%)	6 (3.37%)
**Frequency of agitation, sadness, or anxiety due to the physical distancing measures (*n* = 1936)**			
Never	121 (17.6%)	214 (19.9%)	39 (22.3%)
Some days	1094 (56.5%)	409 (59.6%)	612 (56.9%)
Almost every day	319 (16.5%)	110 (16.0%)	175 (16.3%)
Every day	149 (7.70%)	46 (6.71%)	74 (6.88%)
**COVID-19 vaccine-related influences**			
**Confidence in the COVID-19 vaccines that are being developed (*n* = 1911)**			
Very confident	191 (28.0%)	26 (2.47%)	0 (0.00%)
Confident	424 (62.1%)	453 (43.0%)	12 (6.86%)
Not very confident	61 (8.93%)	493 (46.8%)	31 (17.7%)
Not confident	7 (1.02%)	81 (7.69%)	132 (75.4%)
**Time (*n* = 1943)**			
Before	414 (60.3%)	463 (42.9%)	43 (24.2%)
After	272 (39.7%)	616 (57.1%)	135 (75.8%)

## Data Availability

The data presented in this study are available on request from the corresponding author. The data are not publicly available since this is an ongoing study.

## Supplementary Materials

The following are available online at https://www.mdpi.com/2076-393X/9/3/300/s1, Table S1: Odds of delay and refusal for the determinants of vaccine hesitancy, adjusted for gender, age, education and period of questionnaire. OR: odds-ratio, CI: confidence interval. Table S2: Odds of delay and refusal for the determinants of vaccine hesitancy for individuals without education or with basic or secondary education, adjusted for gender, age, education and period of questionnaire. OR: odds-ratio, CI: confidence interval. Table S3: Odds of delay and refusal for the determinants of vaccine hesitancy for individuals with an university degree, adjusted for gender, age, education and period of questionnaire. OR: odds-ratio, CI: confidence interval. Table S4: Crude odds of delay and refusal for the determinants of vaccine hesitancy. OR: odds-ratio, CI: confidence interval. Table S5: Crude odds of delay and refusal for the determinants of vaccine hesitancy for individuals without education or with basic or secondary education. OR: odds-ratio, CI: confidence interval. Table S6: Crude odds of delay and refusal for the determinants of vaccine hesitancy for individuals with an university degree. OR: odds-ratio, CI: confidence interval.
