# Supplementary material for: Factors Associated with COVID-19 Vaccine Hesitancy
Source: Vaccines (Basel). 2021 Mar 22;9(3):300. doi: 10.3390/vaccines9030300 (PMC8004673; doi:10.3390/vaccines9030300)
Supplement: Supplementary file 1 [file vaccines-09-00300-s001.pdf]

Table S1. Odds of delay and refusal for the determinants of vaccine hesitancy, adjusted for gender, age, education and period of questionnaire. OR: odds-ratio, CI: confidence interval.

	Wait *versus* Yes		No *versus* Yes		No *versus* Wait	
	**OR**	**95% CI**	**OR**	**95% CI**	**OR**	**95% CI**
**Gender** (ref.Male)	**1.44**	**(1.16; 1.78)**	0.91	(0.64; 1.31)	**0.63**	**(0.45; 0.89)**
**Age (in years)**	**0.99**	**(0.98; 0.99)**	**0.98**	**(0.97; 0.99)**	0.99	(0.98; 1.01)
**Education** (ref. University)						
No education/Basic	0.88	(0.50; 1.57)	2.07	(0.92; 4.63)	**2.33**	**(1.08; 5.05)**
Secondary	1.23	(0.96; 1.58)	**1.78**	**(1.19; 2.66)**	1.44	(0.99; 2.10)
**Monthly household income** (ref. <650€)						
651-1000€	1.00	(0.58; 1.72)	0.69	(0.27; 1.71)	0.69	(0.29; 1.61)
1001-1500€	0.98	(0.59; 1.64)	1.10	(0.49; 2.48)	1.11	(0.52; 2.38)
1501-2000€	0.95	(0.57; 1.61)	1.01	(0.43; 2.35)	1.06	(0.48; 2.33)
2001-2500€	1.12	(0.65; 1.91)	1.29	(0.54; 3.08)	1.15	(0.51; 2.59)
> 2501€	0.72	(0.43; 1.20)	0.65	(0.27; 1.52)	0.90	(0.40; 2.02)
**Lost of income due to the pandemic** (ref. No)	**1.26**	**(1.02; 1.57)**	**1.92**	**(1.35; 2.74)**	**1.52**	**(1.09; 2.12)**
**Occupation** (ref. Worker)						
Student	**0.51**	**(0.31; 0.83)**	0.47	(0.19; 1.14)	0.93	(0.40; 2.18)
Unemployed	1.12	(0.69; 1.83)	1.70	(0.84; 3.44)	1.52	(0.80; 2.87)
Retired	0.77	(0.51; 1.18)	**0.19**	**(0.05; 0.64)**	**0.24**	**(0.07; 0.83)**
Other	0.98	(0.60; 1.62)	0.89	(0.38; 2.05)	0.90	(0.41; 1.98)
**Intention of taking the flu vaccine this year** (ref. Yes, I take the flu vaccine every year)						
Yes, I will take the flu vaccine this year	**1.42**	**(1.06; 1.88)**	1.04	(0.34; 3.20)	0.74	(0.24; 2.24)
No	**2.33**	**(1.84; 2.96)**	**19.81**	**(9.74; 40.30)**	**8.50**	**(4.22; 17.11)**
**Perception of the health status** (ref. Very good/Good)						
Reasonable	1.00	(0.81; 1.23)	**0.59**	**(0.40; 0.87)**	**0.59**	**(0.41; 0.86)**
Bad/Very bad	0.71	(0.41; 1.23)	**0.22**	**(0.05; 0.95)**	0.31	(0.07; 1.33)
**Respiratory disease** (ref. No)	1.20	(0.91; 1.58)	1.10	(0.68; 1.78)	0.91	(0.58; 1.44)
**Autoimmune disease** (ref. No)	0.84	(0.61; 1.16)	**0.36**	**(0.16; 0.81)**	**0.43**	**(0.20; 0.95)**
**Number of comorbidities** (ref. 0)						
1	0.92	(0.73; 1.16)	**0.58**	**(0.38; 0.87)**	**0.63**	**(0.42; 0.92)**
≥2	0.97	(0.71; 1.31)	**0.24**	**(0.11; 0.51)**	**0.24**	**(0.12; 0.52)**
**Have school-age children** (ref. No)	1.00	(0.82; 1.22)	**1.93**	**(1.37; 2.73)**	**1.94**	**(1.39; 2.69)**

	Wait *versus* Yes		No *versus* Yes		No *versus* Wait	
**Confidence in the health services capacity to respond to the pandemic** (ref. Very confident)						
Confident	1.28	(0.88; 1.85)	**0.47**	**(0.25; 0.89)**	**0.37**	**(0.20; 0.68)**
Not very confident	**1.69**	**(1.14; 2.51)**	0.92	(0.48; 1.76)	0.54	(0.29; 1.01)
Not confident	1.66	(0.91; 3.06)	**7.56**	**(3.59; 15.92)**	**4.54**	**(2.33; 8.87)**
**View on the information provided by the health authorities** (ref. Clear and understandable)						
Unclear and confusing	1.31	(0.97; 1.77)	**2.44**	**(1.12; 5.29)**	1.86	(0.87; 3.98)
Inconsistent and contradictory	**1.50**	**(1.12; 1.99)**	**8.61**	**(4.73; 15.68)**	**5.75**	**(3.23; 10.26)**
**Perception of the adequacy of the measures implemented by the Government** (ref. Very adequate/adequate)						
Not very adequate/Not adequate	**1.40**	**(1.15; 1.72)**	**8.49**	**(5.44; 13.25)**	**6.04**	**(3.93; 9.30)**
**Self-Perceived Risk to get COVID-19 Infection** (ref. High)						
Moderate	0.92	(0.71; 1.18)	**0.61**	**(0.39; 0.96)**	0.67	(0.44; 1.02)
Low/No risk	1.09	(0.79; 1.49)	**1.98**	**(1.23; 3.20)**	**1.83**	**(1.18; 2.84)**
Not sure	1.13	(0.67; 1.92)	0.38	(0.11; 1.36)	0.34	(0.10; 1.16)
**Self-Perceived Risk to Develop Severe Disease Following COVID-19 Infection** (ref. High)						
Moderate	**1.31**	**(1.00; 1.73)**	1.83	(0.92; 3.64)	1.40	(0.71; 2.74)
Low/No risk	1.27	(0.95; 1.71)	**7.41**	**(3.89; 14.11)**	**5.82**	**(3.10; 10.91)**
Not sure	**1.72**	**(1.17; 2.53)**	1.34	(0.48; 3.73)	0.78	(0.29; 2.12)
**Frequency of agitation, sadness or anxiety due to the physical distance measures** (ref. Never)						
Some days	**0.74**	**(0.57; 0.96)**	**0.49**	**(0.31; 0.78)**	0.67	(0.44; 1.03)
Almost every day	0.74	(0.53; 1.04)	0.82	(0.47; 1.42)	1.10	(0.66; 1.85)
Every day	0.75	(0.48; 1.18)	1.50	(0.81; 2.78)	**2.00**	**(1.13; 3.51)**

	Wait *versus* Yes		No *versus* Yes		No *versus* Wait	
**Confidence in the COVID-19 vaccines that are being developed** (ref. Very confident/Confident)	**9.94**	**(7.48; 13.2)**	**109.69**	**(57.38; 209.69)**	**11.04**	**(6.01; 20.28)**
**Period of questionnaire** (ref. After)	**2.05**	**(1.68; 2.50)**	**4.69**	**(3.21; 6.86)**	**2.29**	**(1.59; 3.30)**

Table S2. Odds of delay and refusal for the determinants of vaccine hesitancy for individuals without education or with basic or secondary education, adjusted for gender, age, education and period of questionnaire. OR: odds-ratio, CI: confidence interval.

	Wait *versus* Yes		No *versus* Yes		No *versus* Wait	
	Multivariate regression		Multivariate regression		Multivariate regression	
	OR	95% CI	OR	95% CI	OR	95% CI
**Gender** (ref.Male)	0.98	(0.63; 1.52)	0.55	(0.29; 1.06)	0.56	(0.31; 1.03)
**Age (in years)**	0.99	(0.97; 1.01)	0.98	(0.95; 1.00)	0.99	(0.96; 1.01)
**Monthly household income** (ref. <650€)						
651-1000€	**0.42**	**(0.18; 0.99)**	**0.20**	**(0.05; 0.74)**	0.47	(0.15; 1.50)
1001-1500€	0.53	(0.23; 1.25)	0.66	(0.20; 2.16)	1.24	(0.46; 3.35)
1501-2000€	**0.28**	**(0.12; 0.68)**	0.58	(0.17; 1.97)	2.07	(0.72; 5.98)
2001-2500€	0.72	(0.26; 2.03)	0.15	(0.01; 1.50)	0.21	(0.02; 1.81)
> 2501€	**0.19**	**(0.07; 0.52)**	**0.15**	**(0.03; 0.93)**	0.82	(0.15; 4.58)
**Lost of income due to the pandemic** (ref. No)	1.19	(0.77; 1.84)	1.57	(0.81; 3.05)	1.32	(0.72; 2.43)
**Occupation** (ref. Worker)						
Student	0.77	(0.28; 2.11)	0.64	(0.13; 3.16)	0.83	(0.19; 3.64)
Unemployed	1.42	(0.65; 3.11)	2.05	(0.72; 5.85)	1.45	(0.59; 3.57)
Retired	1.04	(0.49; 2.20)	0.15	(0.02; 1.31)	0.14	(0.02; 1.22)
Other	0.84	(0.36; 1.98)	1.09	(0.33; 3.62)	1.30	(0.43; 3.93)
**Intention of taking the flu vaccine this year** (ref. Yes, I take the flu vaccine every year)						
Yes, I will take the flu vaccine this year	0.87	(0.46; 1.63)	1.97	(0.12; 33.18)	2.28	(0.14; 38.10)
No	**3.03**	**(1.86; 4.96)**	**65.95**	**(8.64; 503.62)**	**21.73**	**(2.90; 162.76)**
**Perception of the health status** (ref. Very good/Good)						
Reasonable	0.78	(0.51; 1.20)	0.59	(0.30; 1.16)	0.76	(0.41; 1.41)
Bad/Very bad	**0.42**	**(0.18; 0.94)**	**0.11**	**(0.01; 0.92)**	0.27	(0.03; 2.19)
**Respiratory disease** (ref. No)	0.94	(0.54; 1.65)	0.90	(0.36; 2.21)	0.95	(0.41; 2.22)
**Autoimmune disease** (ref. No)	**0.42**	**(0.22; 0.81)**	**0.10**	**(0.01; 0.80)**	0.25	(0.03; 1.91)
**Number of comorbidities** (ref. 0)						

	Wait *versus* Yes		No *versus* Yes		No *versus* Wait	
1	1.06	(0.66; 1.69)	0.59	(0.28; 1.25)	0.56	(0.28; 1.12)
≥2	0.94	(0.52; 1.71)	**0.24**	**(0.06; 0.87)**	**0.25**	**(0.07; 0.89)**
**Have school-age children** (ref. No)	0.88	(0.57; 1.35)	1.36	(0.71; 2.63)	1.54	(0.84; 2.83)
**Confidence in the health services capacity to respond to the pandemic** (ref. Very confident)						
Confident	0.91	(0.44; 1.88)	0.56	(0.17; 1.86)	0.62	(0.20; 1.88)
Not very confident	0.94	(0.43; 2.07)	0.74	(0.20; 2.67)	0.78	(0.24; 2.57)
Not confident	0.40	(0.12; 1.34)	**6.30**	**(1.61; 24.69)**	**15.67**	**(4.17; 58.93)**
**View on the information provided by the health authorities** (ref. Clear and understandable)						
Unclear and confusing	1.25	(0.59; 2.65)	**7.00**	**(1.57; 31.27)**	**5.61**	**(1.34; 23.39)**
Inconsistent and contradictory	1.26	(0.68; 2.35)	**12.81**	**(3.53; 46.44)**	**10.15**	**(2.95; 34.95)**
**Perception of the adequacy of the measures implemented by the Government** (ref. Very adequate/adequate)						
Not very adequate/Not adequate	1.24	(0.81; 1.88)	**14.28**	**(5.23; 38.97)**	**11.55**	**(4.37; 30.52)**
**Self-Perceived Risk to get COVID-19 Infection** (ref. High)						
Moderate	**1.78**	**(1.06; 2.97)**	1.11	(0.44; 2.82)	0.63	(0.26; 1.54)
Low/No risk	**2.38**	**(1.24; 4.56)**	**7.03**	**(2.71; 18.21)**	**2.96**	**(1.24; 7.06)**
Not sure	1.18	(0.50; 2.78)	0.84	(0.15; 4.66)	0.71	(0.13; 3.78)
**Self-Perceived Risk to Develop Severe Disease Following COVID-19 Infection** (ref. High)						
Moderate	**1.74**	**(1.04; 2.94)**	1.99	(0.61; 6.50)	1.14	(0.36; 3.64)
Low/No risk	**2.80**	**(1.52; 5.18)**	**15.06**	**(4.95; 45.82)**	**5.37**	**(1.87; 15.39)**
Not sure	**2.50**	**(1.22; 5.15)**	2.58	(0.53; 12.48)	1.03	(0.23; 4.69)
**Frequency of agitation, sadness or anxiety due to the physical distance measures** (ref. Never)						
Some days	0.67	(0.39; 1.16)	**0.34**	**(0.15; 0.78)**	0.51	(0.24; 1.08)

	Wait *versus* Yes		No *versus* Yes		No *versus* Wait	
Almost every day	0.94	(0.45; 1.97)	0.60	(0.21; 1.74)	0.64	(0.24; 1.66)
Every day	1.08	(0.47; 2.48)	0.75	(0.24; 2.37)	0.70	(0.26; 1.90)
Confidence in the COVID-19 vaccines that are being developed (ref. Very confident/Confident)	**7.37**	**(4.27; 12.74)**	**182.46**	**(39.38; 845.33)**	**24.76**	**(5.67; 108.01)**
**Period of questionnaire** (ref. After)	**1.73**	**(1.15; 2.61)**	**6.86**	**(3.20; 14.70)**	**3.96**	**(1.91; 8.22)**

Table S3. Odds of delay and refusal for the determinants of vaccine hesitancy for individuals with an university degree, adjusted for gender, age, education and period of questionnaire. OR: odds-ratio, CI: confidence interval.

	Wait *versus* Yes		No *versus* Yes		No *versus* Wait	
	Multivariate regression		Multivariate regression		Multivariate regression	
	OR	95% CI	OR	95% CI	OR	95% CI
**Gender** (ref.Male)	**1.63**	**(1.27; 2.09)**	1.10	(0.71; 1.70)	0.68	(0.44; 1.03)
**Age (in years)**	**0.98**	**(0.98; 0.99)**	0.98	(0.97; 1.00)	1.00	(0.98; 1.01)
**Monthly household income** (ref. <650€)						
651-1000€	**2.25**	**(1.07; 4.75)**	2.11	(0.50; 8.84)	0.93	(0.23; 3.79)
1001-1500€	1.82	(0.93; 3.58)	1.96	(0.53; 7.28)	1.07	(0.29; 3.93)
1501-2000€	**2.17**	**(1.09; 4.31)**	1.76	(0.46; 6.79)	0.81	(0.21; 3.07)
2001-2500€	**2.13**	**(1.07; 4.24)**	2.99	(0.8; 11.14)	1.40	(0.38; 5.13)
> 2501€	1.55	(0.80; 3.01)	1.42	(0.39; 5.21)	0.91	(0.25; 3.31)
**Lost of income due to the pandemic** (ref. No)	**1.29**	**(1.01; 1.66)**	**2.08**	**(1.37; 3.16)**	**1.61**	**(1.09; 2.38)**
**Occupation** (ref. Worker)						
Student	**0.44**	**(0.24; 0.80)**	0.41	(0.13; 1.29)	0.93	(0.30; 2.84)
Unemployed	1.00	(0.53; 1.86)	1.55	(0.58; 4.12)	1.56	(0.62; 3.89)
Retired	**0.57**	**(0.33; 0.97)**	**0.22**	**(0.05; 0.99)**	0.38	(0.08; 1.75)
Other	1.12	(0.61; 2.08)	0.70	(0.20; 2.49)	0.63	(0.19; 2.10)
**Intention of taking the flu vaccine this year** (ref. Yes, I take the flu vaccine every year)						
Yes, I will take the flu vaccine this year	**1.61**	**(1.16; 2.22)**	0.91	(0.27; 3.11)	0.57	(0.17; 1.92)
No	**2.21**	**(1.68; 2.90)**	**14.54**	**(6.76; 31.26)**	**6.59**	**(3.1; 14.02)**
**Perception of the health status** (ref. Very good/Good)						
Reasonable	1.09	(0.85; 1.38)	**0.58**	**(0.36; 0.93)**	**0.53**	**(0.33; 0.84)**
Bad/Very bad	0.99	(0.46; 2.14)	0.43	(0.05; 3.37)	0.43	(0.06; 3.35)
**Respiratory disease** (ref. No)	1.31	(0.96; 1.80)	1.18	(0.66; 2.08)	0.90	(0.52; 1.54)
**Autoimmune disease** (ref. No)	1.07	(0.74; 1.56)	0.53	(0.22; 1.28)	0.49	(0.21; 1.16)

	Wait *versus* Yes		No *versus* Yes		No *versus* Wait	
**Number of comorbidities** (ref. 0)						
1	0.90	(0.69; 1.17)	**0.57**	**(0.35; 0.94)**	0.64	(0.40; 1.03)
≥2	0.99	(0.70; 1.41)	**0.23**	**(0.09; 0.61)**	**0.23**	**(0.09; 0.60)**
**Have school-age children** (ref. No)	1.06	(0.84; 1.33)	**2.18**	**(1.44; 3.29)**	**2.06**	**(1.39; 3.05)**
**Confidence in the health services capacity to respond to the pandemic** (ref. Very confident)						
Confident	1.46	(0.95; 2.26)	**0.44**	**(0.21; 0.91)**	**0.30**	**(0.14; 0.62)**
Not very confident	**2.08**	**(1.31; 3.30)**	0.97	(0.46; 2.05)	**0.47**	**(0.22; 0.97)**
Not confident	**2.73**	**(1.31; 5.67)**	**8.21**	**(3.35; 20.13)**	**3.01**	**(1.36; 6.65)**
**View on the information provided by the health authorities** (ref. Clear and understandable)						
Unclear and confusing	1.33	(0.96; 1.86)	1.54	(0.60; 3.95)	1.15	(0.45; 2.93)
Inconsistent and contradictory	**1.56**	**(1.13; 2.15)**	**7.50**	**(3.82; 14.73)**	**4.82**	**(2.50; 9.28)**
**Perception of the adequacy of the measures implemented by the Government** (ref. Very adequate/adequate)						
Not very adequate/Not adequate	**1.46**	**(1.16; 1.84)**	**7.29**	**(4.41; 12.03)**	**4.99**	**(3.07; 8.10)**
**Self-Perceived Risk to get COVID-19 Infection** (ref. High)						
Moderate	0.75	(0.56; 1.00)	**0.50**	**(0.30; 0.82)**	0.66	(0.41; 1.07)
Low/No risk	0.86	(0.60; 1.24)	1.18	(0.67; 2.08)	1.37	(0.81; 2.32)
Not sure	1.26	(0.63; 2.52)	0.24	(0.03; 1.95)	0.19	(0.03; 1.47)
**Self-Perceived Risk to Develop Severe Disease Following COVID-19 Infection** (ref. High)						
Moderate	1.13	(0.82; 1.58)	1.61	(0.69; 3.75)	1.42	(0.62; 3.25)
Low/No risk	0.97	(0.69; 1.37)	**5.34**	**(2.41; 11.85)**	**5.48**	**(2.51; 11.97)**
Not sure	1.40	(0.88; 2.22)	0.88	(0.22; 3.51)	0.63	(0.16; 2.46)
**Frequency of agitation, sadness or anxiety due to the physical distance**						

	Wait *versus* Yes		No *versus* Yes		No *versus* Wait	
**measures** (ref. Never)						
Some days	0.75	(0.55; 1.02)	0.58	(0.33; 1.02)	0.78	(0.46; 1.32)
Almost every day	0.69	(0.47; 1.02)	0.95	(0.49; 1.85)	1.38	(0.74; 2.58)
Every day	0.62	(0.36; 1.06)	**2.12**	**(1.01; 4.45)**	**3.42**	**(1.71; 6.84)**
**Confidence in the COVID-19 vaccines that are being developed** (ref. Very confident/Confident)	**11.55**	**(8.24; 16.20)**	**102.07**	**(49.55; 210.25)**	**8.84**	**(4.52; 17.26)**
**Period of questionnaire** (ref. After)	**2.15**	**(1.71; 2.69)**	**4.00**	**(2.58; 6.20)**	**1.86**	**(1.22; 2.85)**

Table S4. Crude odds of delay and refusal for the determinants of vaccine hesitancy. OR: odds-ratio, CI: confidence interval.

	Wait vs Yes		No vs Yes		No vs Wait	
	OR	95% CI	OR	95% CI	OR	95% CI
**Gender** (ref.Male)	**1.39**	**(1.13; 1.72)**	0.83	(0.59; 1.17)	**0.59**	**(0.43; 0.83)**
**Age (in years)**	**0.98**	**(0.98; 0.99)**	0.98	(0.97; 1.00)	1.00	(0.98; 1.01)
**Education** (ref. University)						
No education/Basic	0.76	(0.43; 1.32)	1.83	(0.86; 3.94)	**2.43**	**(1.15; 5.13)**
Secondary	1.12	(0.88; 1.43)	**1.60**	**(1.09; 2.37)**	1.43	(0.99; 2.06)
**Monthly household income** (ref. <650€)						
651-1000€	1.00	(0.59; 1.70)	0.59	(0.24; 1.44)	0.59	(0.26; 1.36)
1001-1500€	0.86	(0.52; 1.40)	0.74	(0.34; 1.62)	0.87	(0.42; 1.80)
1501-2000€	0.85	(0.51; 1.40)	0.69	(0.31; 1.55)	0.81	(0.38; 1.74)
2001-2500€	0.99	(0.59; 1.66)	0.80	(0.35; 1.83)	0.81	(0.37; 1.75)
> 2501€	**0.58**	**(0.36; 0.94)**	**0.40**	**(0.18; 0.87)**	0.68	(0.32; 1.44)
**Lost of income due to the pandemic** (ref. No)	**1.31**	**(1.06; 1.62)**	**2.04**	**(1.44; 2.87)**	**1.56**	**(1.12; 2.15)**
**Occupation** (ref. Worker)						
Student	0.77	(0.50; 1.17)	0.72	(0.33; 1.56)	0.94	(0.44; 2.01)
Unemployed	1.16	(0.72; 1.86)	1.86	(0.95; 3.63)	1.61	(0.87; 2.97)
Retired	**0.52**	**(0.36; 0.74)**	**0.15**	**(0.05; 0.47)**	**0.28**	**(0.09; 0.92)**
Other	1.07	(0.66; 1.73)	1.06	(0.47; 2.38)	0.99	(0.46; 2.14)
**Intention of taking the flu vaccine this year** (ref. Yes, I take the flu vaccine every year)						
Yes, I will take the flu vaccine this year	**1.58**	**(1.20; 2.08)**	1.11	(0.37; 3.38)	0.71	(0.24; 2.16)
No	**2.40**	**(1.92; 3.01)**	**18.29**	**(9.16; 36.53)**	**7.66**	**(3.84; 15.24)**
**Perception of the health status** (ref. Very good/Good)						
Reasonable	0.95	(0.78; 1.16)	**0.56**	**(0.39; 0.82)**	**0.59**	**(0.42; 0.85)**

	Wait vs Yes		No vs Yes		No vs Wait	
Bad/Very bad	**0.57**	**(0.34; 0.97)**	**0.21**	**(0.05; 0.88)**	0.36	(0.08; 1.53)
**Respiratory disease** (ref. No)	1.17	(0.90; 1.53)	1.02	(0.64; 1.64)	0.87	(0.56; 1.37)
**Autoimmune disease** (ref. No)	0.85	(0.63; 1.16)	**0.32**	**(0.15; 0.72)**	**0.38**	**(0.17; 0.83)**
**Number of comorbidities** (ref. 0)						
1	0.87	(0.70; 1.08)	**0.58**	**(0.39; 0.85)**	**0.66**	**(0.46; 0.96)**
≥2	0.85	(0.64; 1.13)	**0.23**	**(0.11; 0.48)**	**0.27**	**(0.13; 0.56)**
**Have school-age children** (ref. No)	1.04	(0.85; 1.26)	**1.95**	**(1.39; 2.73)**	**1.88**	**(1.36; 2.59)**
**Confidence in the health services capacity to respond to the pandemic** (ref. Very confident)						
Confident	1.33	(0.92; 1.90)	**0.48**	**(0.26; 0.87)**	**0.36**	**(0.20; 0.65)**
Not very confident	**1.82**	**(1.24; 2.68)**	1.00	(0.53; 1.86)	0.55	(0.30; 1.01)
Not confident	1.79	(0.99; 3.25)	**8.63**	**(4.21; 17.70)**	**4.82**	**(2.50; 9.30)**
**View on the information provided by the health authorities** (ref. Clear and understandable)						
Unclear and confusing	1.24	(0.93; 1.66)	**2.25**	**(1.05; 4.83)**	1.82	(0.86; 3.86)
Inconsistent and contradictory	**1.58**	**(1.20; 2.08)**	**8.67**	**(4.85; 15.5)**	**5.49**	**(3.12; 9.65)**
**Perception of the adequacy of the measures implemented by the Government** (ref. Very adequate/adequate)						
Not very adequate/Not adequate	**1.44**	**(1.18; 1.76)**	**9.34**	**(6.02; 14.48)**	**6.46**	**(4.22; 9.91)**
**Self-Perceived Risk to get COVID-19 Infection** (ref. High)						
Moderate	0.84	(0.66; 1.08)	**0.54**	**(0.35; 0.83)**	**0.64**	**(0.42; 0.97)**
Low/No risk	0.93	(0.68; 1.25)	**1.58**	**(1.00; 2.50)**	**1.71**	**(1.11; 2.63)**
Not sure	0.97	(0.58; 1.63)	0.34	(0.10; 1.17)	0.35	(0.10; 1.17)
**Self-Perceived Risk to Develop Severe Disease Following COVID-19 Infection** (ref. High)						
Moderate	**1.34**	**(1.02; 1.75)**	1.64	(0.84; 3.21)	1.22	(0.63; 2.38)
Low/No risk	**1.44**	**(1.10; 1.89)**	**6.60**	**(3.60; 12.1)**	**4.57**	**(2.52; 8.31)**

	Wait vs Yes		No vs Yes		No vs Wait	
Not sure	**1.77**	**(1.21; 2.58)**	1.24	(0.45; 3.42)	0.70	(0.26; 1.90)
**Frequency of agitation, sadness or anxiety due to the physical distance measures** (ref. Never)						
Some days	0.85	(0.66; 1.09)	**0.55**	**(0.36; 0.86)**	**0.65**	**(0.43; 0.99)**
Almost every day	0.90	(0.65; 1.25)	0.96	(0.57; 1.62)	1.07	(0.65; 1.76)
Every day	0.91	(0.59; 1.40)	**1.96**	**(1.09; 3.52)**	**2.15**	**(1.24; 3.72)**
**Confidence in the COVID-19 vaccines that are being developed** (ref. Very confident/Confident)	**10.84**	**(8.21; 14.32)**	**122.85**	**(64.94; 232.4)**	**11.34**	**(6.23; 20.63)**
**Time** (ref. After)	**2.05**	**(1.68; 2.50)**	**4.69**	**(3.21; 6.86)**	**2.36**	**(1.64; 3.39)**

Table S5. Crude odds of delay and refusal for the determinants of vaccine hesitancy for individuals without education or with basic or secondary education. OR: odds-ratio, CI: confidence interval.

	Wait vs Yes		No vs Yes		No vs Wait	
	OR	95% CI	OR	95% CI	OR	95% CI
**Gender** (ref.Male)	0.97	(0.63; 1.49)	**0.53**	**(0.28; 0.98)**	**0.55**	**(0.30; 0.98)**
**Age (in years)**	0.99	(0.98; 1.01)	0.98	(0.96; 1.00)	0.99	(0.97; 1.01)
**Monthly household income** (ref. <650€)						
651-1000€	**0.43**	**(0.18; 0.99)**	**0.2**	**(0.05; 0.72)**	0.47	(0.15; 1.45)
1001-1500€	0.48	(0.21; 1.13)	0.51	(0.16; 1.58)	1.05	(0.41; 2.73)
1501-2000€	**0.26**	**(0.11; 0.62)**	0.44	(0.14; 1.39)	1.69	(0.61; 4.65)
2001-2500€	0.68	(0.24; 1.89)	0.11	(0.01; 1.08)	0.17	(0.02; 1.41)
> 2501€	**0.17**	**(0.06; 0.47)**	**0.12**	**(0.02; 0.68)**	0.69	(0.13; 3.69)
**Lost of income due to the pandemic** (ref. No)	1.24	(0.81; 1.90)	1.73	(0.92; 3.28)	1.40	(0.77; 2.54)
**Occupation** (ref. Worker)						
Student	0.97	(0.46; 2.02)	0.77	(0.23; 2.49)	0.79	(0.26; 2.44)
Unemployed	1.44	(0.66; 3.13)	1.99	(0.73; 5.42)	1.38	(0.58; 3.32)
Retired	0.78	(0.43; 1.42)	**0.11**	**(0.01; 0.83)**	0.14	(0.02; 1.05)
Other	0.90	(0.39; 2.08)	1.24	(0.40; 3.87)	1.38	(0.47; 4.04)
**Intention of taking the flu vaccine this year** (ref. Yes, I take the flu vaccine every year)						
Yes, I will take the flu vaccine this year	0.89	(0.48; 1.63)	2.13	(0.13; 35.19)	2.39	(0.14; 39.61)
No	**3.04**	**(1.92; 4.83)**	**72.07**	**(9.63; 539.10)**	**23.62**	**(3.20; 174.26)**
**Perception of the health status** (ref. Very good/Good)						
Reasonable	0.78	(0.51; 1.18)	**0.51**	**(0.27; 0.97)**	0.66	(0.36; 1.20)
Bad/Very bad	**0.41**	**(0.19; 0.90)**	**0.11**	**(0.01; 0.88)**	0.27	(0.03; 2.17)

	Wait vs Yes		No vs Yes		No vs Wait	
**Respiratory disease** (ref. No)	0.95	(0.55; 1.64)	0.84	(0.36; 2.00)	0.89	(0.39; 2.03)
**Autoimmune disease** (ref. No)	**0.44**	**(0.23; 0.84)**	**0.10**	**(0.01; 0.73)**	0.22	(0.03; 1.67)
**Number of comorbidities** (ref. 0)						
1	1.08	(0.69; 1.71)	0.63	(0.31; 1.28)	0.58	(0.30; 1.13)
≥2	0.91	(0.51; 1.61)	**0.23**	**(0.07; 0.82)**	**0.26**	**(0.08; 0.88)**
**Have school-age children** (ref. No)	0.99	(0.65; 1.49)	1.63	(0.88; 3.04)	1.66	(0.92; 2.98)
**Confidence in the health services capacity to respond to the pandemic** (ref. Very confident)						
Confident	0.85	(0.41; 1.74)	0.46	(0.14; 1.46)	0.54	(0.18; 1.61)
Not very confident	0.94	(0.43; 2.06)	0.68	(0.20; 2.38)	0.72	(0.23; 2.33)
Not confident	0.40	(0.12; 1.33)	**6.07**	**(1.66; 22.12)**	**15.00**	**(4.15; 54.27)**
**View on the information provided by the health authorities** (ref. Clear and understandable)						
Unclear and confusing	1.31	(0.63; 2.72)	**7.69**	**(1.82; 32.45)**	**5.89**	**(1.47; 23.57)**
Inconsistent and contradictory	1.45	(0.80; 2.63)	**12.38**	**(3.66; 41.92)**	**8.54**	**(2.64; 27.66)**
**Perception of the adequacy of the measures implemented by the Government** (ref. Very adequate/adequate)						
Not very adequate/Not adequate	1.29	(0.85; 1.95)	**15.83**	**(5.96; 42.02)**	**12.26**	**(4.74; 31.72)**
**Self-Perceived Risk to get COVID-19 Infection** (ref. High)						
Moderate	**1.69**	**(1.02; 2.80)**	1.02	(0.42; 2.52)	0.61	(0.25; 1.46)
Low/No risk	**2.09**	**(1.11; 3.95)**	**5.83**	**(2.38; 14.32)**	**2.79**	**(1.20; 6.47)**
Not sure	1.04	(0.45; 2.39)	0.67	(0.13; 3.46)	0.64	(0.12; 3.27)
**Self-Perceived Risk to Develop Severe Disease Following COVID-19 Infection** (ref. High)						
Moderate	**1.72**	**(1.03; 2.85)**	1.94	(0.61; 6.16)	1.13	(0.36; 3.55)
Low/No risk	**2.75**	**(1.56; 4.86)**	**15.45**	**(5.50; 43.37)**	**5.61**	**(2.08; 15.14)**
Not sure	**2.35**	**(1.15; 4.80)**	2.24	(0.48; 10.45)	0.95	(0.21; 4.25)

	Wait vs Yes		No vs Yes		No vs Wait	
**Frequency of agitation, sadness or anxiety due to the physical distance measures** (ref. Never)						
Some days	0.73	(0.43; 1.24)	0.41	(0.19; 0.89)	0.56	(0.28; 1.16)
Almost every day	1.08	(0.53; 2.22)	0.83	(0.30; 2.26)	0.76	(0.31; 1.91)
Every day	1.18	(0.53; 2.62)	1.08	(0.37; 3.15)	0.91	(0.35; 2.39)
**Confidence in the COVID-19 vaccines that are being developed** (ref. Very confident/Confident)	**7.04**	**(4.19; 11.81)**	**159.55**	**(36.25; 702.21)**	**22.67**	**(5.40; 95.11)**
**Period of questionnaire** (ref. After)	**1.73**	**(1.15; 2.61)**	**7.29**	**(3.42; 15.52)**	**4.21**	**(2.03; 8.69)**

Table S6. Crude odds of delay and refusal for the determinants of vaccine hesitancy for individuals with an university degree. OR: odds-ratio, CI: confidence interval.

	Wait vs Yes		No vs Yes		No vs Wait	
	OR	95% CI	OR	95% CI	OR	95% CI
**Gender** (ref.Male)	**1.59**	**(1.25; 2.03)**	1.04	(0.68; 1.59)	**0.65**	**(0.43; 0.99)**
**Age (in years)**	**0.98**	**(0.97; 0.99)**	0.98	(0.96; 1.00)	1.00	(0.98; 1.01)
**Monthly household income** (ref. <650€)						
651-1000€	**2.17**	**(1.05; 4.48)**	1.80	(0.44; 7.40)	0.83	(0.21; 3.34)
1001-1500€	1.55	(0.81; 2.99)	1.49	(0.41; 5.45)	0.96	(0.27; 3.50)
1501-2000€	1.88	(0.97; 3.66)	1.38	(0.37; 5.22)	0.73	(0.20; 2.75)
2001-2500€	1.80	(0.92; 3.51)	2.24	(0.61; 8.16)	1.25	(0.34; 4.50)
> 2501€	1.16	(0.62; 2.20)	1.04	(0.29; 3.72)	0.90	(0.25; 3.20)
**Lost of income due to the pandemic** (ref. No)	**1.33**	**(1.05; 1.70)**	**2.13**	**(1.41; 3.21)**	**1.60**	**(1.08; 2.36)**
**Occupation** (ref. Worker)						
Student	0.67	(0.39; 1.14)	0.59	(0.20; 1.72)	0.88	(0.30; 2.55)
Unemployed	0.99	(0.54; 1.84)	1.41	(0.54; 3.65)	1.42	(0.57; 3.50)
Retired	**0.40**	**(0.25; 0.63)**	**0.16**	**(0.04; 0.66)**	0.40	(0.09; 1.69)
Other	1.13	(0.62; 2.07)	0.70	(0.20; 2.44)	0.62	(0.19; 2.06)
**Intention of taking the flu vaccine this year** (ref. Yes, I take the flu vaccine every year)						
Yes, I will take the flu vaccine this year	**1.79**	**(1.31; 2.46)**	0.98	(0.29; 3.33)	0.55	(0.16; 1.85)
No	**2.24**	**(1.73; 2.89)**	**12.69**	**(6.04; 26.68)**	**5.67**	**(2.71; 11.85)**
**Perception of the health status** (ref. Very good/Good)						
Reasonable	0.99	(0.79; 1.25)	**0.51**	**(0.32; 0.82)**	**0.52**	**(0.33; 0.81)**
Bad/Very bad	0.69	(0.33; 1.44)	0.26	(0.03; 1.97)	0.37	(0.05; 2.86)

	Wait vs Yes		No vs Yes		No vs Wait	
**Respiratory disease** (ref. No)	1.26	(0.93; 1.70)	1.10	(0.63; 1.92)	0.87	(0.51; 1.49)
**Autoimmune disease** (ref. No)	1.05	(0.73; 1.51)	0.47	(0.20; 1.13)	0.45	(0.19; 1.05)
**Number of comorbidities** (ref. 0)						
1	0.81	(0.63; 1.04)	**0.55**	**(0.34; 0.87)**	0.67	(0.43; 1.06)
≥2	0.83	(0.60; 1.15)	**0.22**	**(0.08; 0.55)**	**0.26**	**(0.10; 0.65)**
**Have school-age children** (ref. No)	1.06	(0.85; 1.32)	**2.14**	**(1.43; 3.20)**	**2.03**	**(1.37; 2.99)**
**Confidence in the health services capacity to respond to the pandemic** (ref. Very confident)						
Confident	**1.58**	**(1.04; 2.42)**	**0.48**	**(0.23; 0.98)**	**0.30**	**(0.15; 0.62)**
Not very confident	**2.29**	**(1.46; 3.60)**	1.12	(0.54; 2.33)	0.49	(0.24; 1.01)
Not confident	**3.02**	**(1.48; 6.19)**	**9.63**	**(4.02; 23.1)**	**3.19**	**(1.46; 6.98)**
**View on the information provided by the health authorities** (ref. Clear and understandable)						
Unclear and confusing	1.26	(0.91; 1.73)	1.48	(0.58; 3.78)	1.17	(0.46; 2.98)
Inconsistent and contradictory	**1.63**	**(1.20; 2.22)**	**7.81**	**(4.03; 15.14)**	**4.80**	**(2.52; 9.12)**
**Perception of the adequacy of the measures implemented by the Government** (ref. Very adequate/adequate)						
Not very adequate/Not adequate	**1.50**	**(1.20; 1.88)**	**7.72**	**(4.71; 12.67)**	**5.16**	**(3.18; 8.36)**
**Self-Perceived Risk to get COVID-19 Infection** (ref. High)						
Moderate	**0.68**	**(0.51; 0.91)**	**0.44**	**(0.27; 0.72)**	0.65	(0.40; 1.04)
Low/No risk	0.72	(0.50; 1.02)	0.92	(0.53; 1.59)	1.28	(0.76; 2.15)
Not sure	1.08	(0.55; 2.12)	0.20	(0.02; 1.56)	0.18	(0.02; 1.39)
**Self-Perceived Risk to Develop Severe Disease Following COVID-19 Infection** (ref. High)						
Moderate	1.19	(0.87; 1.64)	1.58	(0.68; 3.64)	1.32	(0.58; 3.02)
Low/No risk	1.20	(0.88; 1.64)	**5.38**	**(2.51; 11.53)**	**4.48**	**(2.11; 9.51)**
Not sure	1.52	(0.97; 2.38)	0.87	(0.22; 3.45)	0.57	(0.15; 2.22)

	Wait vs Yes		No vs Yes		No vs Wait	
**Frequency of agitation, sadness or anxiety due to the physical distance measures** (ref. Never)						
Some days	0.88	(0.66; 1.18)	0.65	(0.38; 1.12)	0.74	(0.44; 1.25)
Almost every day	0.86	(0.59; 1.24)	1.11	(0.59; 2.10)	1.30	(0.70; 2.39)
Every day	0.77	(0.46; 1.29)	**2.57**	**(1.26; 5.25)**	**3.34**	**(1.69; 6.59)**
**Confidence in the COVID-19 vaccines that are being developed** (ref. Very confident/Confident)	**12.63**	**(9.06; 17.62)**	**114.92**	**(56.24; 234.86)**	**9.10**	**(4.69; 17.64)**
**Period of questionnaire** (ref. After)	**2.12**	**(1.70; 2.65)**	**4.04**	**(2.61; 6.25)**	**1.91**	**(1.25; 2.91)**
